# DOPA-Responsive Tremor Associated With Gammopathy: A Case Report and Literature Review

**DOI:** 10.7759/cureus.19963

**Published:** 2021-11-28

**Authors:** Saeed Hamidi, Mona Kafaie, Ulviyya Gasimova

**Affiliations:** 1 Neurology, Essentia Health Hospital, Fargo, USA; 2 Biology, Saint Louis University, St. Louis, USA; 3 Neurology, Saint Louis University School of Medicine, St. Louis, USA

**Keywords:** lambda paraproteinemia, dopamine repletion, gammopathy, parkinsonism, tremor

## Abstract

Tremors have been well-described in association with monoclonal gammopathy. We report a case of a patient with asymmetric hands tremor who responded well to levodopa-carbidopa treatment. Further workup showed an underlying gammopathy. To our knowledge, this is one of the rarest case reports of successful treatment of gammopathy-related tremors with levodopa-carbidopa. The patient was a 75-year-old male who presented to the neurology clinic for a one-year history of worsening tremors in bilateral upper extremities. A review of systems was only remarkable for mild numbness and tingling in his feet. His neurological examination was remarkable for asymmetric right more than left (R > L) resting and kinetic tremor in both upper extremities associated with mild rigidity and bradykinesia in right upper extremity and decreased bilateral ankle jerks. With the primary diagnosis of Parkinson’s disease, he was started on levodopa-carbidopa and a neuropathy workup was requested. His follow-up visit after three months was remarkable for significant improvement of his tremors with carbidopa-levodopa. However, his blood work was consistent with a significant increase in lambda light chain levels and the presence of an M spike in serum protein electrophoresis. Based on the presentation and clinical workup, he was finally found to have both multiple myeloma and ​Waldenstrom’s macroglobulinemia. Underlying malignancy was treated with chemotherapy and immunotherapy. Levodopa-carbidopa was discontinued after three months of chemotherapy and his tremor did not recur in one year of follow-up. Gammopathy is one of the well-known causes of tremors in the adult population. It can cause both resting and kinetic tremors in the upper extremities. It is supposed that peripheral neuropathy associated with gammopathy is the main underlying cause of tremors in these groups of patients. However, central causes are also suggested. In this case, we are led to conclude that our patient’s tremor was centrally mediated since it responded well to dopamine replacement therapy. However, further study is needed to elucidate the role of dopamine depletion in the pathogenesis of tremors associated with gammopathies.

## Introduction

Monoclonal gammopathy can present with systemic symptoms such as fatigue, generalized weakness, weight loss as well as anemia, bleeding, and increased bruising. This condition may lead to the involvement of the nervous system, be it the central or peripheral nervous system, causing changes in mental status, headache, visual changes, peripheral neuropathies, etc. Monoclonal gammopathy-associated tremors have been well-described [1]. Gammopathy is one of the well-known causes of tremors in the adult population. It can cause both resting and kinetic tremors in the upper extremities. It is supposed that peripheral neuropathy associated with gammopathy is the main underlying cause of tremors in these groups of patients. However, central causes are also suggested.

## Case presentation

The patient is a 75-year-old Caucasian, right-handed male who presented with bilateral hand shakiness. His tremors had started one year ago but had gotten worse during the last two months. The tremor was noted to be more severe in his right hand. He was a professional chess player, but he had recently developed difficulty playing chess and difficulty with handwriting due to tremors. He had a remote history of right hip trauma for which he underwent surgery, but it was complicated with shortening of the right femur and limping, and his gait had become more unsteady during the last two months, which resulted in using a cane. His hand tremors were both at rest and with movements.

Review of systems was remarkable for limping and also numbness and tingling in his feet. It was negative for double vision, blurred vision, drooling, ​dysphonia or dysphagia, memory impairment, hallucination, mood change, disinhibition, agitation, depression, anxiety, hands or limbs weakness, constipation, changes in smell, urinary incontinence, abnormal ​movements or kicking during sleep, recent falls and hearing problems, family or personal history of essential tremor or Parkinson’s disease, and drinking excessive coffee or alcohol. His past medical history was remarkable for chronic kidney disease (CKD) stage III and well-controlled type 2 diabetes mellitus for 10-15 years on oral hypoglycemic agents complicated by diabetic neuropathy. His neuropathic symptoms such as numbness and tingling in his feet have started five years ago and used to be very mild -1/10 in severity, and he had never been on any medication for his mild neuropathic pain. However, he had noticed some worsening of his numbness and tingling for the last two months and also worsening in his limping, and he started using a cane at the same time. He was never a smoker and drinker. He was able to drive and was independent with simple and complex activities of daily life.

Positive findings in his neurological examination were pill-rolling resting tremor (more pronounced in the right hand), with a frequency of 6-8 Hz, which was enhanced with mental distraction and ​contralateral voluntary movements, mild to moderate postural and action tremor of bilateral arms, with mild rigidity and bradykinesia noted in right hand, decreased ankle jerks bilaterally, and mildly decreased light touch and pinprick sensation in both feet up to distal legs. He walked with a cane and his gait was stable but slow with limping due to remote history of right hip surgery, and he had severe genu varum in both knees. During walking, arms swing was observed but a significant tremor was noted in the right hand that was holding a cane. His handwriting had been impaired due to tremors.

With an initial diagnosis of neuropathic tremor versus parkinsonism, after discussion with the patient, he was started on a trial of carbidopa-levodopa (C/L) 25/100 mg three times a day (TID). Due to the recent worsening of his numbness and tingling and also mild anemia and hemoglobin A1C (HbA1C) at 7.5 in reviewing of his labs, a neuropathy workup was requested. A follow-up visit after two months was remarkable for significant improvement in his stiffness, gait, and tremor, especially in the morning. However, he​ complained of some tremors in the afternoon around 3-4 pm. It was found to be an off symptom due to taking C/L at 8 am, 5 pm, and one at midnight. No side ​effects or intolerance with this medication were reported. Blood workup was remarkable for normocytic normochromic anemia (hemoglobin level of 8 gm/dL), creatinine of 1.4, and HbA1c of 7.7. Other significant laboratory abnormalities were elevated M spike in serum protein electrophoresis (SPEP), the elevation of immunoglobulin M (IgM) level almost five times of normal value, and a significant increase in kappa/lambda light chain ratio (more than 18 times of normal value) (Table 1). Other labs (serum vitamin B12, methylmalonic acid (MMA), comprehensive metabolic panel, vitamin D3 (OH)25, free thyroxine, and thyroid-stimulating hormone) were within normal ranges. The patient was recommended to continue C/L, and he was also referred to physical therapy and follow up with hematology-oncology clinic for management of his underlying gammopathy. He was also recommended to adjust the timing of taking C/L to 7 am, 12 pm, and 5 pm.

**Table 1:** Abnormalities in the laboratory results: IgM-lambda paraproteinemia. IgG, immunoglobulin G; IgA, immunoglobulin A; IgM, immunoglobulin M.

Immunoglobulins/kappa/lambda	Values in our patient	Normal values
IgG	676 mg/L	520-1822 mg/L
IgA	131 mg/L	63-484 mg/L
IgM	1134 mg/L	22-240 mg/L
Lambda free	507 mg/L	4.2-27.7mg/L
Kappa free	25.6 mg/L	2.4-20.7 mg/L

Further oncology workup including ​bone marrow biopsy was consistent with 80% involvement with B-cell lymphoma associated with IgM monoclonal protein in ​serum consistent with the combination of lymphoplasmacytic lymphoma (LPL) and Waldenstrom’s macroglobulinemia (WM). The genetic test was positive for MYD88 L265P point mutation. No evidence of myelodysplastic syndrome, ​atypical lymphocytes, or metastatic adenocarcinoma was found. Infiltrate was positive for CD20, lambda, IgM, and MYD88 but negative for CD5 and CD10. Myelin-associated glycoprotein (MAG) antibodies and Congo red staining for amyloid were negative as well. He received two injections of bendamustine and rituximab for multiple myeloma (MM) and WM. He continued taking C/L during oncology workup and his chemotherapy/immunotherapy and tremors remained well-controlled with a near-complete improvement of tremor and his balance. A follow-up visit in nine months from the initial presentation was remarkable for the complete improvement of tremors and stiffness. He stopped taking C/L by himself since he thought that it made him feel stiff in his ​hands in the morning. However, no recurrence or reemergent of tremors was observed in the next three and six-month follow-ups while being off the C/L. His gammopathy was well-controlled with treatment. His neurological examination in almost one year of his initial presentation was notable for no resting or action tremor, subtle fine postural tremor was found immediately in outstretched hands, as well as no rigidity, and his gait was very fast and stable without a cane; however, he was still limping due to the remote history of right hip shortening.

## Discussion

We reported on a 75-year-old Caucasian male who presented with a one-year history of asymmetric, slowly progressive, and distally predominant resting and kinetic tremor in bilateral upper extremities with mild rigidity and bradykinesia, who responded very well to C/L 25/100 mg TID. Due to the presence of mild numbness and tingling in both feet, a workup for ruling out underlying peripheral neuropathy was done, and he was found to have MM (lambda paraproteinemia) and WM. His C/L continued during diagnostic workup for gammopathy and his both resting and kinetic tremor disappeared even before treatment of his underlying gammopathy. He stopped C/L after the second cycle of treatment of underlying gammopathy due to complete improvement of his tremors and feeling stiff while taking medication with no recurrence of his resting or kinetic tremor after at least one year of follow-up.

We have considered four differentials that were suggested for the pathophysiology of our patient’s tremors: neuropathic tremor, paraneoplastic parkinsonism, a combination of both etiologies, and alternative causes of tremor. Our first differential was a neuropathic tremor, given the presence of kinetic, postural, and resting tremor and mild numbness and tingling in his feet. However, the presence of mild rigidity and bradykinesia were against this diagnosis as the only underlying etiology for our patient’s neurological symptoms and signs. Tremor is one of the presenting or accompanying symptoms of peripheral neuropathies especially in demyelinating-predominant neuropathies such as chronic inflammatory demyelinating polyneuropathy (CIDP) or gammopathies. Neuropathic tremors are usually kinetic and postural. Tremor is more prominent in distal extremities but is also present in proximal arms [1]. In gammopathy-related tremors, the frequency in hand muscles can be lower than in proximal arm muscles. In addition, it shares some features of essential tremor (ET) including frequency and amplitude at the beginning, but it has less chronicity, usually less than one year, and is more irregular or jerky than ET [1]. Resting tremor has also been reported with neuropathic tremor but with a lower amplitude than parkinsonian tremor or ET [1]. Less frequently we can see some jerky or pseudoathetosis movements or abduction/adduction patterns with neuropathic tremors. It also may cause ataxia that may mimic parkinsonian unsteadiness.

Tremor increases the disability in patients with inflammatory neuropathies and usually responds poorly to treatment unless the underlying condition is cured early. In one study, tremor was observed most commonly in IgM paraproteinemic neuropathies but was also seen in 58% of patients with CIDP and 56% of patients with multifocal motor neuropathy (MMN) with conduction block. Why tremor occurs only in a subset of these patients, but not all is unclear [2]. Available data suggest no clear relationship between tremor and severity of the accompanying neuropathy as assessed by proprioceptive loss, weakness, or fatigue [3,4]. Conduction velocity does not correlate with tremor emergence [4] although this may influence its severity [3]. However, Saifee et al. demonstrated for the first time that in patients with MMN tremor, severity correlates with F wave latency [2]. They also showed that tremors may add to disability in patients with inflammatory neuropathy. The mean tremor frequency was 6 Hz and did not vary with weight loading.

Ahlskog et al. compared tremor occurrence in IgM-monoclonal gammopathy of undetermined significance (MGUS) cases to a control group and found that tremor occurrence was significantly higher in the IgM-MGUS case cohort than in the control cohort [5]. In the treatment group, demyelinating-type neuropathy was the most common finding; however, 18% of cases had axonal neuropathy. The tremor was also associated with a worse neuropathy impairment score (NIS). Postural-kinetic tremors were the most common type (81.6%) in IgM-MGUS, with resting tremors reported in 13%, and in 5% with mixed resting-action tremors. Alternative causes of tremor were identified in 42% of IgM-MGUS cases, and the most common type was inherited ET 6/60. They concluded that among IgM-MGUS neuropathy cases, severity and type of neuropathy (demyelinating over axonal) are correlated with tremor occurrence [5].

IgM paraproteinemia (PIgM) is a common cause of tremors. Irregular and symmetrical tremors involving the distal and proximal parts of limbs were described by Bain et al. in patients with PIgM [6]. Upper limb tremors in 16 patients with PIgM were also described by Smith et al. [7]. Among them, seven cases had parkinsonian tremors and five patients were described to have additional kinetic lower limb tremors. Tremors in patients with IgM-MGUS were also described by Ahlskog et al. [5].

Our second differential was paraneoplastic parkinsonism, given a presentation with predominantly resting tremor, presence of mild rigidity and bradykinesia, lack of prominent neuropathic symptoms, and significant improvement of his symptoms with C/L. However, kinetic tremors and numbness and tingling in his feet were against this diagnosis as the sole underlying etiology.

Paraneoplastic parkinsonism is very rare. One of the first cases was reported by Golbe et al. in 1989 [8]. The patient was a 42-year-old woman who presented with rapidly progressive parkinsonism and painful hands dystonia. She was found to have invasive carcinoma of the breast with extracranial metastasis. Her initial symptoms included 4-5 Hz course action tremor of all limbs, more pronounced at elbows with lower amplitude at rest. Other parkinsonian symptoms were included difficulty initiating gait, wide-based and slow gait with mild unsteadiness, and myoclonic jerks with minimal rest tremor. Her symptoms progressed rapidly after four days and developed severe dysarthria and dysphagia, and painful dystonia. She was found to have invasive carcinoma of the breast with extracranial metastasis. Her parkinsonism symptoms did not respond to C/L, anticholinergics, baclofen, diazepam, and plasmapheresis. No autoantibodies were found in the serum of the patient, cerebrospinal fluid (CSF) study and electroencephalogram (EEG) were normal, and computer tomography (CT) of the head with contrast was unremarkable. She passed away four months later, and her autopsy revealed loss of substantia nigra neurons and cerebellar Purkinje cells. The putamen was normal and stains for anti-human immunoglobulin G (IgG), IgM, kappa, and lambda were negative.

Tada et al. reported a 72-year-old man who presented with gradual progressive difficulty with walking, sialorrhea, stooped posture, and short stepped gait [9]. Later he developed dysarthria and the inability to walk unassisted. Marked bradykinesia, decreased arms swing, and facial masking was found, but no rigidity or tremor was observed. His workup was remarkable for bilateral T2 and fluid-attenuated inversion recovery (FLAIR) hyperintensities in the lenticular and caudate nuclei in brain magnetic resonance imaging (MRI), high concentrations of progastrin-releasing peptide and neuron-specific enolase in serum, a small lung nodule, and swollen lymph nodes in the mediastinum. Lymph node biopsy was compatible with small cell lung cancer. In addition, serum and CSF paraneoplastic panel were positive only for high titer of anti-collapsin response mediator protein 5 (anti-CRMP5) antibodies. His gait freezing did not respond to levodopa, but his gait and MRI abnormalities improved after chemotherapy and radiotherapy and gradually became ambulatory with an assistant. No immunotherapy was applied.

Nabil et al. also reported a 63-year-old woman with severe paraneoplastic parkinsonism who initially presented with gradual worsening of resting tremor for six months, action tremor, difficulty with walking, and unsteadiness for three months [10]. The resting tremor was asymmetric in the beginning but later became bilateral. Her symptoms worsened gradually, and she developed bradykinesia, rigidity, unsteadiness with frequent falls, hyperreflexia on the right side, impairment of downward gaze, slow saccades, and personality changes. Non-specific white matter hyperintensities were seen in T2 MRI images in the caudate and putamen bilaterally without midbrain atrophy. Workup revealed increased CSF IgG index, and serum/CSF paraneoplastic panels were positive for high concentrations of anti-Ri antibodies. Whole-body positron emission tomography (PET) showed an active lesion on the left breast and its biopsy revealed a high-grade infiltrating ductal carcinoma. No local or regional metastasis was found. Therapy with levodopa was tried while waiting for paraneoplastic results but her motor symptoms were refractory to treatment. The patient was diagnosed with the definite paraneoplastic neurological syndrome (PNS) but did not respond clinically to a five-day course of 1 gr daily intravenous (IV) methylprednisolone. Then, she received quadrantectomy and ipsilateral axillary lymphadenectomy, and adjuvant chemotherapy; however, the patient died eight months later.

A 63-year-old woman with atypical parkinsonism with the frontal syndrome was presented by Endres et al. [11]. At the age of 60, her symptoms started with insomnia and, in a year, progressed into personality changes with depression and delusions followed by severe cognitive impairment and postural unsteadiness, and parkinsonian symptoms in the left upper extremity. Brain MRI was consistent with mild mesencephalon atrophy and fluorodeoxyglucose (FDG) PET scan showed moderate hypometabolism in bilateral frontal lobes and the midbrain. Severely reduced dopamine transporter availability in both striata, indicating pronounced nigrostriatal degeneration, was noted in single-photon emission computed tomography (SPECT). Serum paraneoplastic panel was remarkable for high titer (1:640) of anti-glycine receptor (anti-GlyR) antibodies but it was negative in CSF. No clinical improvement was reported with steroids and azathioprine, but treatment was accompanied by a decrease in antibody titers (to 1:80). In addition, due to the lack of other symptoms of autoimmune anti-GlyR antibodies syndrome such as hyperexcitability and having normal CSF studies, they presumed this finding to be only an unrelated bystander. They also presumed that the anti-GlyR antibodies might have developed secondarily to neurodegeneration, without overt clinical effects. Therefore, such antibodies might have the potential to modify the clinical course of classical movement disorders.

Parkinsonian symptoms have been also reported associated with anti-IgLON5 encephalopathy. Gaig et al. evaluated the clinical manifestations of the anti-IgLON5 disease in 22 patients retrospectively [12]. Anti-IgLON5 encephalopathy may present initially with four different clinical pictures including parasomnia and sleep apnea, bulbar syndrome, chorea with cognitive decline, and a progressive supranuclear palsy-like syndrome. However, all of them will end up with sleep disorders and parasomnia. IgLON5 antibodies (more common IgG1 and IgG4 subclasses) were found in either serum or CSF or both. Parkinsonian symptoms, most commonly gait abnormalities (36% of cases), and mild bradykinesia or rigid-akinetic parkinsonism may present during the course of the disease. They suggested multiple causes for gait problems including subcortical origin in about half of the patients due to severe gait abnormality accompanied by impaired balance and abnormal postural reflexes. Dysfunction of mesencephalic nuclei, which are involved in the control of gait and balance, were proposed as pathophysiology underlying this type of gait dysfunction. Routine MRI studies did not show clear atrophy in those patients, but autopsy studies demonstrated the involvement of the midbrain tegmentum. Twenty of those patients were treated with immunotherapy, which usually included monthly cycles of IV steroids or immunoglobulins. Only two patients had mild and transient improvement of symptoms, and 13 patients (59%) died.

Lastly, Bing-Neel syndrome (BNS) has been also reported as a very rare complication of WM due to lymphoplasmacytoid infiltration and IgM deposition in the CNS [13]. It has two distinct forms: tumoral and diffuse infiltrative. The neurologic presentation tends to develop over a period of weeks to months and can be extremely variable, depending on the location and extension of the lesions. Parenchymal involvement can present with seizures, weakness, aphasia, memory deficits, psychiatric symptoms, or even coma. In meningeal forms, patients usually present with cranial nerves palsies, headache, and vomiting. Sensory deficits and weakness are suggestive of spinal cord involvement. However, no pure parkinsonian syndrome has been reported with this syndrome.

## Conclusions

We proposed that underlying MM (lambda paraproteinemia) and WM were causative factors for the development of both neuropathic tremors and paraneoplastic parkinsonian syndrome in our patient. To our knowledge, this case is one of the rarest reports of dopamine responsive tremors associated with gammopathy. The patient’s parkinsonian symptoms responded well to dopamine replacement therapy even before treatment of underlying gammopathy. The patient remained symptom-free after successful and early treatment of underlying gammopathies with four cycles of chemotherapy/immunotherapy and discontinuation of C/L. The presence of concurrent mild numbness and tingling of feet led to comprehensive neuropathy workup, which ended up with an early diagnosis and early treatment of the abovementioned gammopathies. We could not find other causes for his parkinsonian symptoms. The initial diagnosis of Parkinson’s disease was wrong since he remained symptom-free even after discontinuation of dopamine replacement therapy. The drawback of our study is that our only evidence for the presence of dopa-responsive paraneoplastic parkinsonian features was clinical. His brain MRI without contrast was normal. No CSF study was done to show if CSF had positive inflammatory indexes or to show the presence of one of the pathologic neuronal autoantibodies. The other argument is that if we should have sent paraneoplastic workup for all patients with parkinsonian features. The answer would be no. We think we need to rule out underlying autoimmune/paraneoplastic parkinsonism if the patient presents with atypical parkinsonian features including the bilateral onset of symptoms, rapid progression of the symptoms, early unsteadiness, presence of concomitant neuropathic symptoms like numbness and tingling or weakness, lack of prodromal symptoms of Parkinson’s disease such as difficulty smelling, rapid eye movement (REM) behavior disorder, and constipation, presence of risk factor for malignancy including older age, and constitutional symptoms such as weight change. Finally, gammopathies are a relatively common finding in older age and one of the main culprits for causing neuropathic tremors in the older population. Further study is needed to determine the incidence of paraneoplastic/autoimmune parkinsonism.
